# Nomograms Based on Blood-Based Biomarkers for Predicting Prognosis in Locally Advanced Nasopharyngeal Carcinoma Patients

**DOI:** 10.1155/mi/6618728

**Published:** 2025-03-10

**Authors:** Sisi Wang, Yuhua Feng, Jie Ling, Xiayan Zhao, Yanming Hu, Tao Hou, Yangchun Xie

**Affiliations:** ^1^Department of Oncology, The Second Xiangya Hospital of Central South University, Changsha, Hunan, China; ^2^Department of Oncology and Hematology, Turpan City People's Hospital, Turpan, Xinjiang, China

**Keywords:** locally advanced nasopharyngeal carcinoma, nomogram, platelet-to-platelet distribution width ratio, prognosis, systemic inflammation response index

## Abstract

**Purpose:** This study aimed to investigate the prognostic significance of the platelet-to-platelet distribution width ratio (P/PDW), systemic inflammation response index (SIRI), and systemic immune inflammation index (SII) in patients with locally advanced nasopharyngeal carcinoma (LA-NPC).

**Methods:** A total of 549 LA-NPC patients were included in this retrospective analysis. Clinicopathological characteristics and blood test data were obtained from patient records. Receiver operating characteristic (ROC) curve analysis was employed to determine the optimal cutoff values for P/PDW, SIRI, and SII. The *χ*^2^ test was used to compare clinicopathological characteristics. Survival rates were calculated using the Kaplan–Meier method. Prognostic factors were evaluated using univariate and multivariate analyses via Cox regression. Additionally, we developed a nomogram to predict outcomes and assessed its acuracy using the concordance index (C-index) and a calibration curve.

**Results:** The median follow-up time was 47.1 months. Elevated P/PDW levels were associated with advanced N stages and higher risks of disease progression (all *p*  < 0.05). Patients with high SIRI or SII levels were more likely to have advanced T stages, clinical stages, and to develop metastasis (all *p*  < 0.05). Univariate analysis revealed that P/PDW, SIRI, SII, and T stage were significantly correlated with both overall survival (OS) and progression-free survival (PFS; all *p*  < 0.05). Clinical stage was significantly related only to PFS (*p*=0.009). Multivariate Cox regression analysis identified P/PDW (hazard ratio (HR): 0.544, 95% confidence interval (CI): 0.390–0.759, *p*  < 0.001; HR: 0.406, 95% CI: 0.268–0.615, *p*  < 0.001) and T stage (HR: 0.539, 95% CI: 0.378–0.768, *p*=0.001; HR: 0.545, 95% CI: 0.364–0.815, *p*=0.003) as independent prognostic factors for both OS and PFS, while SIRI (HR: 0.525, 95% CI: 0.333–0.827, *p*=0.006) was an independent predictor of OS. Nomogram C-indexes for the nomogram of OS were 0.717 and PFS were 0.711, respectively. Survival predictions and actual survival were consistent according to the calibration curve.

**Conclusion:** Our findings suggest that P/PDW is a convenient and effective marker for predicting outcomes in LA-NPC patients.

## 1. Introduction

Nasopharyngeal carcinoma (NPC) is a prevalent head and neck malignancy in Southern China and Southeast Asia, originating from the nasopharyngeal epithelium [1]. The preferred treatment for locoregionally advanced NPC is radiotherapy combined with chemotherapy [2], while targeted therapy and immunotherapy have shown promise for recurrent or metastatic cases [3]. Due to the lack of early symptoms and appropriate screening tools, most NPC patients are diagnosed at advanced stages, resulting in poor prognosis. Reports indicate that approximately 14% of NPC patients experience relapse and 21% suffer from metastasis [4], with only 63%–78% surviving for 5 years [5, 6]. Therefore, identifying prognostic factors is crucial for patient stratification and therapeutic guidance.

Increasing evidence suggests that inflammation, a critical hallmark of cancer, significantly impacts tumorigenesis and progression [7]. Activated immune and inflammatory cells, such as neutrophils, lymphocytes, monocytes, and platelets, may promote tumor growth and development by inducing DNA damage or interfering with DNA repair [8, 9]. Systemic inflammation response index (SIRI) and systemic immune inflammation index (SII), based on peripheral blood cell counts, have been shown to predict the prognosis of various cancers, including colorectal cancer [10, 11], pancreatic cancer [12, 13], cervical cancer [14, 15], and ovarian cancer [16, 17]. Platelets, key regulators of cancer-associated inflammation, have been proven to accelerate tumor growth and metastasis in colorectal cancer [18] and oral cancer [19]. Platelet distribution width (PDW), an indicator of platelet volume variation, is closely associated with the diagnosis and prognosis of laryngeal, liver, and colorectal cancers [20–22]. Combining platelet count with PDW may provide a more effective prognostic marker for malignant tumors. To our knowledge, this study is the first to investigate the prognostic value of the platelet-to-platelet distribution width ratio (P/PDW) in locally advanced NPC (LA-NPC) patients.

The present study aims to explore the prognostic value of P/PDW, SIRI, and SII in a retrospective cohort of 549 LA-NPC patients. Additionally, we constructed a nomogram to aid in predicting overall survival (OS) and progression-free survival (PFS) for these patients based on P/PDW values.

## 2. Materials and Methods

### 2.1. Patients Selection

Patients pathological diagnosed as NPC from December 1st, 2013 to December 1st, 2020 were collected at the Second Xiangya Hospital, Central South University in Changsha, China. The following were the inclusion criteria: (1) pathological diagnosis of nasopharyngeal squamous cell carcinoma; (2) stage III–IV according to the 8th edition of the American Joint Committee on Cancer/Union for International Cancer Control (AJCC/UICC) staging system for NPC; (3) receiving treatment at the Second Xiangya Hospital, Central South University; (4) blood test was taken within 1 week prior to the treatment, and all the clinical data were available. The following were the exclusion criteria: (1) with other malignant tumors or chronic inflammatory diseases; (2) with recent steroid therapy; (3) with recent clinical evidence of acute infection or inflammation. Consequently, the study enrolled a total of 549 patients and obtained approval from the Ethics Committee of the Second Xiangya Hospital of Central South University, and informed consent was waived.

### 2.2. Data Collection

The clinicopathological characteristics including age, gender, T stage, N stage, clinical stage, disease status, and Eastern Cooperative Oncology Group performance status (ECOG PS) scores were collected from the electronic medical record system of the Second Xiangya Hospital. Blood routine test within 1 week before therapy were also obtained from the patient's record. The P/PDW, SIRI, and SII were calculated according to the following formulas: P/PDW = platelet count/PDW, SIRI = neutrophil count × monocyte count/lymphocyte count, SII = platelet count × neutrophil count/lymphocyte count. The ECOG PS was a method of patients' functional status evaluation, which had a scale of 0–5. As the ECOG PS score rise, the patients become more restricted in function. The OS was calculated from the date of diagnosis to the date of death for any reason or last follow-up. The PFS was calculated from the date of diagnosis to the date of disease progression based on response evaluation criteria in solid tumors (RECIST) 1.1, or death. All patients were routinely followed up every 3 months. The last follow-up date was July 28, 2024. Model performance and TNM stage were assessed using Harrell's concordance index (C-index).

### 2.3. Statistical Analysis

In this study, SPSS version 22.0 (SPSS Inc., Chicago, IL, USA.) and R software (version 4.1.3) were used for statistical analysis. *χ*^2^ test was used to analyze the relationship between P/PDW, SIRI, SII, and clinicopathological factors in LA-NPC patients. Receiver operating characteristic (ROC) curves were used to determine the optimal cutoff value for P/PDW, SIRI, and SII. Kaplan–Meier method was used to calculate the survival rates, and log-rank test was used to compare the differences between the survival curves. Multivariate Cox hazard regression analysis was used to find out independent predictive factors for survival. All tests were two-sided and *p* values less than 0.05 were statistically significant. Additionally, a nomogram was developed to illustrate the predictive power of the index for OS and PFS.

## 3. Results

### 3.1. Patient Characteristics

In this study, we included 549 patients with LA-NPC. The clinicopathological characteristics are summarized in Table 1. The median age of the cohort was 49 years (range: 19–81 years), with 445 (70.7%) male patients. Regarding T stage, 239 (43.5%) patients were classified as T1 or T2, while 310 (56.5%) were T3 or T4. In terms of N stage, 66 (12.0%) patients were N0 or N1, and 483 (88.0%) were N2 or N3. The distribution of clinical stages showed that 400 (72.9%) patients were at stage III and 149 (27.1%) at stage IV. For the ECOG PS, 266 (48.5%) patients had a score of 0 and 283 (51.5%) had a score of 1–2. The median follow-up duration was 47.1 months (range: 8.8–97.3 months). By the final follow-up, 115 (20.9%) patients had died, and 153 (27.9%) had experienced disease progression.

### 3.2. Cutoff Values of P/PDW, SIRI, SII, and the Correlation With Clinicopathological Characteristics

**Figure 1:**
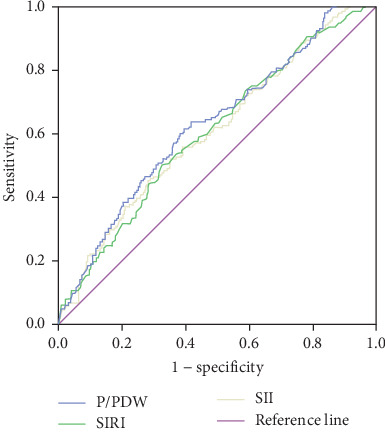
ROC curve analysis for optimal cutoff value of P/PDW, SIRI, and SII for PFS. P/PDW, platelet-to-platelet distribution width ratio; PFS, progression-free survival; ROC, receiver operating characteristic; SII, systemic immune inflammation index; SIRI, systemic inflammation response index.

The optimal cutoff values for P/PDW, SIRI, and SII were determined using ROC curve analysis for PFS. As depicted in Figure 1, the area under the curve (AUC) values for P/PDW, SIRI, and SII were 0.626, 0.603, and 0.606, respectively. The optimal cutoff values were identified as 18.63 for P/PDW, 0.97 for SIRI, and 733.44 for SII.  Table 2 outlines the relationships between P/PDW, SIRI, SII, and various clinicopathological features. Elevated P/PDW levels were significantly associated with later N stages and increased risks of disease progression (all *p*  < 0.05). Patients with higher SIRI or SII values were more likely to present with advanced T stages, higher clinical stages, and a greater likelihood of metastasis (all *p*  < 0.05). However, no significant correlations were found between P/PDW, SIRI, and SII with age, gender, or ECOG PS scores (all *p*  > 0.05).

### 3.3. Univariate and Multivariate Cox Regression Analysis for OS and PFS

**Figure 2:**
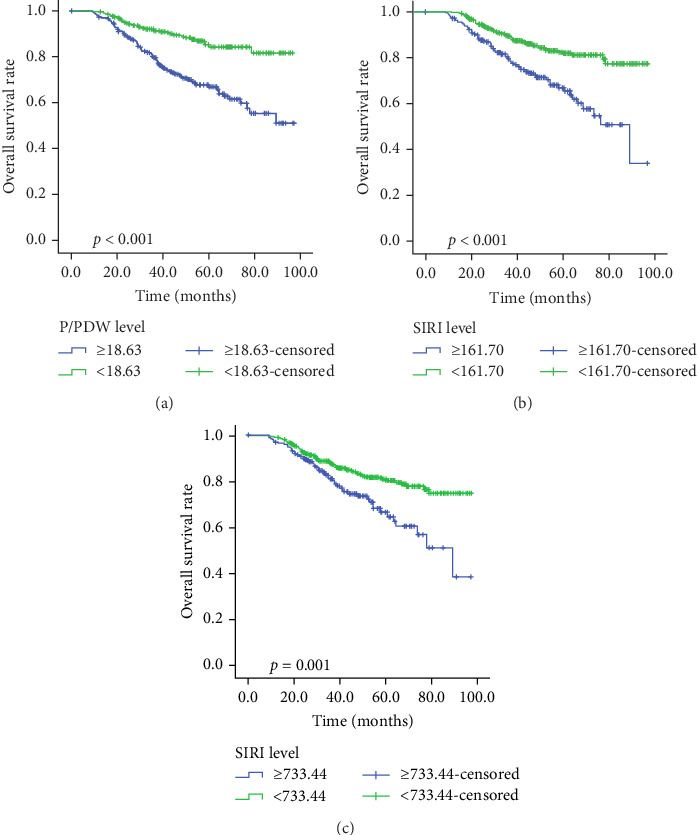
Kaplan–Meier survival curves for OS according to P/PDW (A), SIRI (B), and SII (C) in LA-NPC patients. LA-NPC, locally advanced nasopharyngeal carcinoma; OS, overall survival; P/PDW, platelet-to-platelet distribution width ratio; SII, systemic immune inflammation index; SIRI, systemic inflammation response index.

**Figure 3:**
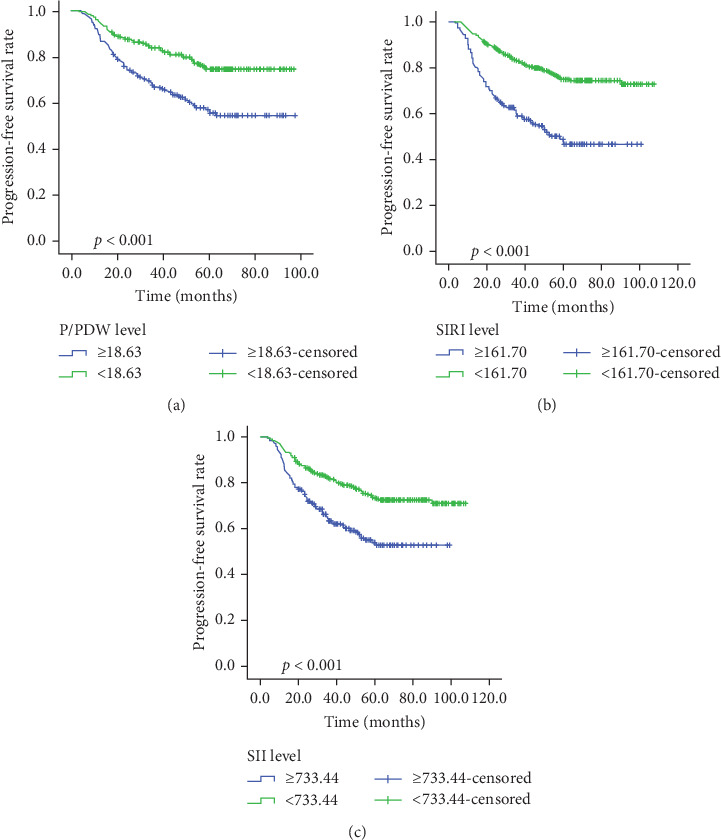
Kaplan–Meier survival curves for PFS according to P/PDW (A), SIRI (B), and SII (C) in LA-NPC patients. LA-NPC, locally advanced nasopharyngeal carcinoma; P/PDW, platelet-to-platelet distribution width ratio; PFS, progression free survival; SII, systemic immune inflammation index; SIRI, systemic inflammation response index.

Kaplan–Meier survival curves for OS and PFS, stratified by P/PDW, SIRI, and SII, are illustrated in Figures 2 and 3. Analysis revealed that P/PDW, SIRI, SII, and T stage were significant predictors of both OS and PFS (*p*  < 0.05, Table 3). Clinical stage was significantly associated with PFS only (*p*=0.009, Table 3). Other clinical characteristics, including age, gender, N stage, and ECOG PS, did not show statistically significant associations in this analysis (Table 3).

A multivariate Cox regression model, incorporating P/PDW, SIRI, SII, T stage, and clinical stage, identified P/PDW (hazard ratio (HR): 0.544, 95% confidence interval (CI): 0.390–0.759, *p*  < 0.001; HR: 0.406, 95% CI: 0.268–0.615, *p*  < 0.001) and T stage (HR: 0.539, 95% CI: 0.378–0.768, *p*=0.001; HR: 0.545, 95% CI: 0.364–0.815, *p*=0.003) as independent prognostic factors for both OS and PFS. Additionally, SIRI (HR: 0.525, 95% CI: 0.333–0.827, *p*=0.006) emerged as an independent predictor of OS in LA-NPC patients (Table 4).

### 3.4. Nomogram

We created innovative nomograms to estimate OS and PFS in patients with NPC, integrating novel and traditional prognostic indicators. The OS nomogram combined key variables, such as T stage (T1-2 vs. T3-4), N stage (N0-1 vs. N2-3), and the P/PDW (P/PDW ≤18.63 vs. P/PDW >18.63), assigning scores based on their relative impact on patient outcomes. A higher cumulative score corresponded to a worse prognosis.

**Figure 4:**
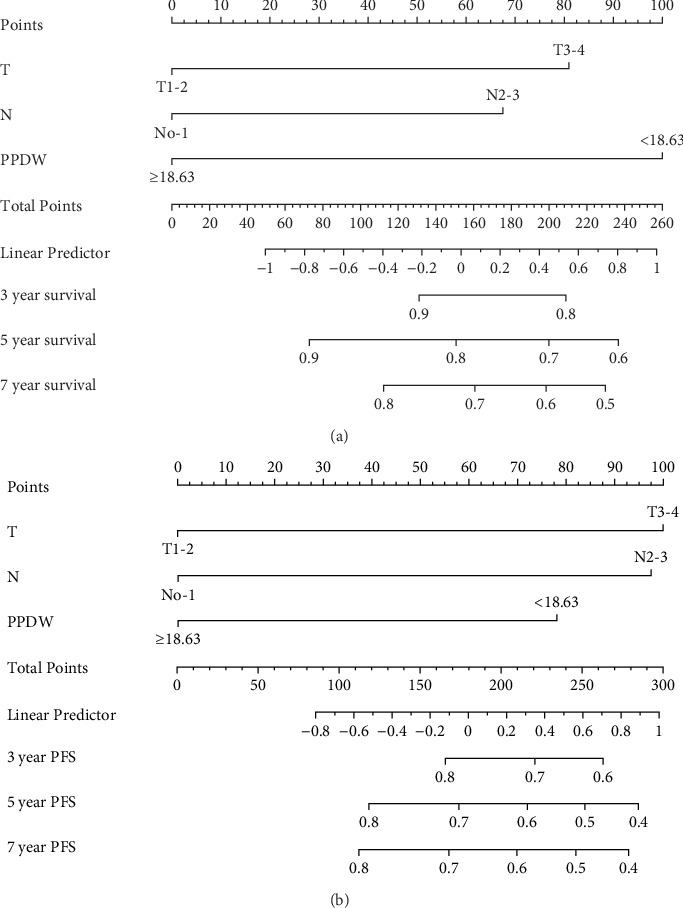
Nomogram for OS (A) and PFS (B) predicting at 3, 5, and 7 years. OS, overall survival; PFS, progression-free survival.

Each predictor's line segment in the nomogram illustrated its relative influence on NPC survival, with P/PDW demonstrating a particularly significant impact. This underscores P/PDW's potential as a critical prognostic marker for OS and PFS in NPC patients, alongside traditional clinicopathological factors (Figure 4).

**Figure 5:**
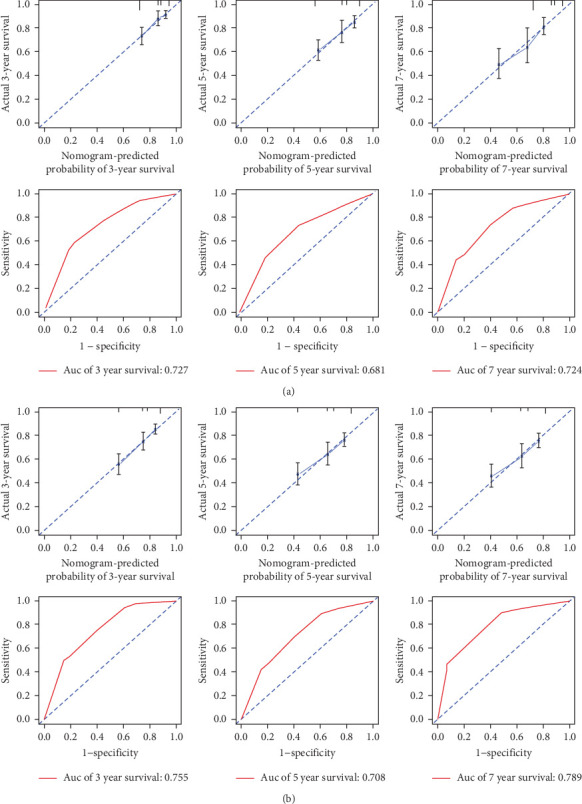
The nomogram-predicted probablity of 3 years OS, 5 years OS, 7 years OS, and the AUCs for 3 years OS, 5 years OS, 7 years OS (A). The nomogram-predicted probablity of 3 years PFS, 5 years PFS, 7 years PFS, and the AUCs for 3 years PFS, 5 years PFS, 7 years PFS (B). AUC, area under the curve; OS, overall survival; PFS, progression-free survival.

To ensure the nomogram's robustness and prevent overfitting, we conducted bootstrap validation with 2000 resamples. Calibration plots for predicting 3-, 5-, and 7-year OS (Figure 5) demonstrated a strong correlation between predicted and observed outcomes for both OS and PFS across cohorts. This validation confirms the nomogram's reliability and precision in forecasting 3-, 5-, and 7-year survival probabilities in NPC patients.

### 3.5. Assessment of Model Performance

Our evaluation of model performance yielded compelling results, underscoring the robustness of the nomogram. ROC curve analysis revealed the model's discriminative power, with ROC values of 0.727, 0.681, and 0.724 for predicting 3-, 5-, and 7-year survival rates, respectively, in the studied cohorts (Figure 5). Notably, the integration of the P/PDW with traditional clinicopathological parameters significantly enhanced the predictive accuracy, as demonstrated by the improved ROC curves in both the training and validation sets.

Furthermore, we assessed predictive performance using the C-index. The OS cohort exhibited a C-index of 0.717, while the PFS nomogram achieved a C-index of 0.711. Importantly, the proposed nomogram consistently demonstrated a C-index exceeding 0.71 for predicting both OS and PFS across cohorts. This indicates its superior predictive efficacy when compared to the 8th edition TNM staging system.

### 3.6. Comparison of Predictive Accuracy for OS and PFS Between Nomogram and TNM Staging System

**Figure 6:**
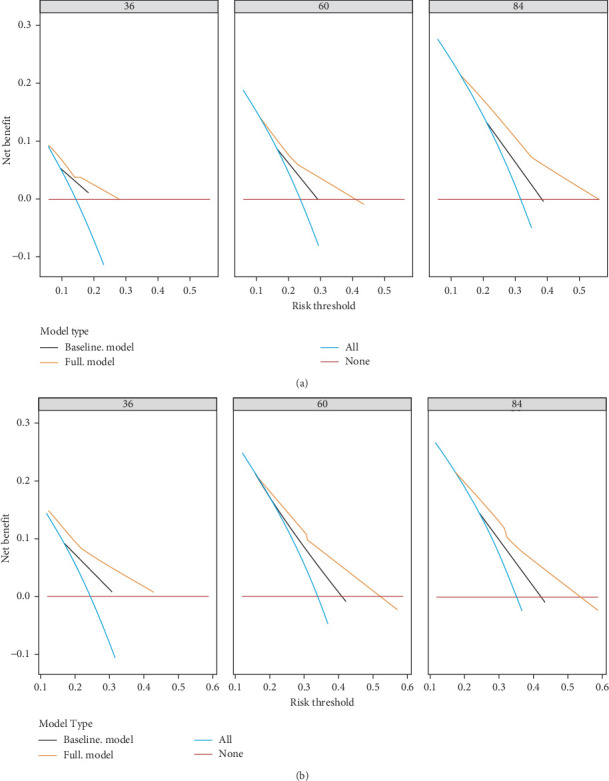
The calibration curves for 3 years OS, 5 years OS, 7 years OS (A), and 3 years PFS, 5 years PFS, 7 years PFS (B). OS, overall survival; PFS, progression-free survival.

To evaluate the practicality of our nomogram, we employed decision curve analysis (DCA), which revealed an optimal threshold range of approximately 10%–50% (Figure 6). Within this threshold range, the nomogram exhibited superior predictive accuracy compared to both the “full model” and “baseline model” approaches for OS and PFS. This enhanced performance was particularly evident in the nomogram's ability to provide a broader range of threshold probabilities, thereby increasing its clinical utility.

The findings from DCA emphasize the nomogram's improved efficacy over the traditional 8th TNM staging system in predicting OS and PFS. The wider range of effective threshold probabilities indicates that the nomogram offers a more flexible and accurate tool for clinical decision-making, thereby facilitating better patient stratification and individualized treatment planning.

## 4. Discussion

In this study, we investigated the prognostic implications of the P/PDW, an immune-inflammation index, in a cohort of 549 patients diagnosed with LA-NPC. Our analysis revealed that elevated P/PDW levels independently predict poorer outcomes in terms of both OS and PFS among LA-NPC patients. A nomogram incorporating P/PDW and other clinical parameters was created, exhibiting strong predictive performance and accuracy, as evidenced by its C-index value and calibration curve. This suggests that P/PDW could be a valuable blood-based immune-inflammation biomarker for forecasting LA-NPC patient prognosis.

In cancer pathology, inflammation serves as a pivotal driver influencing tumor progression through various mechanisms. Tumor cells themselves stimulate systemic inflammation by secreting pro-inflammatory factors, thereby inducing alterations in lymphocyte, neutrophil, monocyte, and platelet counts [23]. Lymphocytes, crucial mediators of antitumor immunity, produce cytokines such as INF-*γ* and TNF-*α*, facilitating tumor cell apoptosis and influencing overall immune competence [24]. Consequently, reduced peripheral blood lymphocyte levels are associated with compromised immunity and poorer prognostic outcomes [25, 26]. Within the tumor microenvironment, neutrophils release immunoregulatory substances that promote cancer progression and evade immune surveillance [23, 27]. Monocytes, another critical component, differentiate into tumor-associated macrophages (TAMs), facilitating tumor infiltration and metastasis [28]. Elevated serum monocyte levels reflect TAM activity, further influencing cancer aggressiveness. Platelets, essential for coagulation, also play a significant role in tumor angiogenesis via the vascular endothelial growth factor (VEGF) pathway and contribute to tumor cell proliferation and metastasis through cytokine release [29]. Increasing evidence underscores the potential of blood-based inflammatory biomarkers as prognostic indicators across various cancers. Combined indices like SIRI and SII, integrating neutrophil, lymphocyte, monocyte, and platelet counts, have shown promise in predicting cancer outcomes.

Previous studies in NPC have highlighted the prognostic significance of SIRI and SII. Feng et al. [30] reported that lower SII (<488.90) and SIRI (<0.86) were associated with prolonged OS and PFS in a cohort of 417 NPC patients, with SIRI identified as an independent predictor for both endpoints (*p*  < 0.05). Similarly, other investigations have linked elevated SII (>804.08) and SIRI (>1.34) with decreased OS and PFS rates in NPC [31]. Furthermore, a retrospective analysis confirmed SII as an independent predictor for 3-year and 5-year survival in NPC, surpassing other inflammation markers like PLR, NLR, and MLR [32]. In our current study, we found that SIRI independently correlated with OS, whereas SII did not emerge as a significant prognostic factor for either OS or PFS, which partially aligns with previous NPC research. This disparity may stem from variations in patient demographics and differing cutoff values applied across studies.

In addition to its association with inflammatory processes, P/PDW has garnered attention for its potential prognostic role in various cancers [20–22]. Cytokines like IL-6, G-CSF, and M-CSF not only influence tumor metastasis and angiogenesis, but also regulate bone marrow cell function and platelet size, underscoring PDW's potential as a cancer outcome indicator [33, 34]. Platelets themselves serve as crucial inflammatory markers and have been established as effective predictors of prognosis across diverse cancer types. For instance, in NPC, elevated PDW (>16.3 fl) and increased platelet count (>266 × 10^9^/L) were significantly linked to poorer prognosis in a cohort study involving 168 patients [35]. Similarly, research in oral cancer by Demir and Abuzaid [36] found higher PDW (>16.65 fl) and platelet count (>281 × 10^9^/L) associated with reduced survival rates. Combining platelet count and PDW into the novel indicator P/PDW may enhance reliability in predicting cancer outcomes. However, the specific prognostic role of P/PDW in LA-NPC patients remains unexplored, and our study represents the first to establish P/PDW as an independent predictor of prognosis in this context. A nomogram was constructed that confirmed the accuracy of the P/PDW prediction.

While our findings underscore P/PDW's promise in predicting outcomes in LA-NPC, several limitations warrant consideration. First, our study was retrospective and conducted at a single center without external validation, potentially limiting generalizability. Second, variations in cutoff values for P/PDW, SIRI, and SII across different studies hinder direct comparisons of outcomes. Third, other prognostic indicators such as EBV-DNA [37], CRP [38], LDH [39], and D-dimer [40] were not included due to incomplete data, potentially introducing bias into our results. Future prospective studies incorporating multiple centers are essential to validate the prognostic value of P/PDW in LA-NPC patients comprehensively.

## 5. Conclusion

In summary, P/PDW is a convenient, cost-effective, and reliable prognostic indicator independently linked to both OS and PFS in LA-NPC patients. Future well-designed prospective studies are essential to validate its prognostic significance and elucidate its underlying mechanisms.

## Figures and Tables

**Table 1:** Clinicopathological characteristics of 549 patients.

Characteristics	Number (%)
Age	
Median	49
Range	19–80
Gender	
Female	156 (28.4)
Male	393 (71.6)
T stage	
1-2	239 (43.5)
3-4	310 (56.5)
N stage	
0-1	66 (12.0)
2-3	483 (88.0)
Clinical stage	
III	400 (72.9)
IV	149 (27.1)
Recurrence	
Yes	62 (11.3)
No	487 (88.7)
Metastasis	
Yes	106 (19.3)
No	443 (80.7)
ECOG PS	
0	266 (48.5)
1–2	283 (51.5)
P/PDW	
<18.63	285 (51.9)
≥18.63	264 (48.1)
SIRI	
<0.97	342 (62.3)
≥0.97	207 (37.7)
SII	
<733.44	367 (66.8)
≥733.44	182 (33.2)

Abbreviations: ECOG PS, Eastern Cooperative Oncology Group performance status; N, nodal; P/PDW, platelet-to-platelet distribution width ratio; SII, systemic immune inflammation index; SIRI, systemic inflammation response index; T, tumor.

**Table 2:** The association of P/PDW, SIRI, and SII with clinicopathologic characteristics in 549 patients with LA-NPC.

Characteristics	P/PDW (<18.63)	P/PDW (≥18.63)	*p*	SIRI (<0.97)	SIRI (≥0.97)	*p*	SII (<733.44)	SII (≥733.44)	*p*
Age									
<65	265 (93.0%)	244 (92.4%)	0.801	322 (94.2%)	187 (90.3%)	0.096	343 (93.5%)	166 (91.2%)	0.339
≥65	20 (7.0%)	20 (7.6%)	—	20 (5.8%)	20 (9.7%)	—	24 (6.5%)	16(8.8%)	—
Gender									
Female	76 (26.7%)	80 (30.3%)	0.345	106 (31.0%)	50 (24.2%)	0.085	104 (28.3%)	52 (28.6%)	0.954
Male	209 (73.3%)	184 (69.7%)	—	236 (69.0%)	157 (75.8%)	—	263 (71.7%)	130 (71.4%)	—
T stage									
1-2	132 (46.3%)	107 (40.5%)	0.172	166 (48.5%)	73 (35.3%)	**0.002**	173 (47.1%)	66 (36.3%)	**0.016**
3-4	153 (53.7%)	157 (59.5%)	—	176 (51.5%)	134 (64.7%)	—	194 (52.9%)	116 (63.7%)	—
N stage									
0-1	43 (15.1%)	23 (8.7%)	**0.022**	39 (11.4%)	27 (13.0%)	0.567	42 (11.4%)	24 (13.2%)	0.554
2-3	242 (84.9%)	241 (91.3%)	—	303 (88.6%)	180 (87.0%)	—	325 (88.6%)	158 (86.8%)	—
Clinical stage									
III	212 (74.4%)	188 (71.2%)	0.403	263 (76.9%)	137 (66.2%)	**0.006**	280 (76.3%)	120 (65.9%)	**0.010**
IV	73 (25.6%)	76 (28.8%)	—	79 (23.1%)	70 (33.8%)	—	87 (23.7%)	62 (34.1%)	—
Recurrence									
Yes	24 (8.4%)	38 (14.4%)	**0.027**	35 (10.2%)	27 (13.0%)	0.313	38 (10.4%)	24 (13.2%)	0.324
No	261 (91.6%)	226 (85.6%)	—	307 (89.8%)	180 (87.0%)	—	329 (89.6%)	158 (86.8%)	—
Metastasis									
Yes	38 (13.3%)	68 (25.8%)	**＜0.001**	49 (14.3%)	57 (27.5%)	**＜0.001**	56 (15.3%)	50 (27.5%)	**0.001**
No	247 (86.7%)	196 (74.2%)	—	293 (85.7%)	150 (72.5%)	—	311 (84.7%)	132 (72.5%)	—
ECOG PS									
0	135 (47.4%)	131 (49.6%)	0.598	176 (51.5%)	90 (43.5%)	0.070	178 (48.5%)	88 (48.4%)	0.974
1–2	150 (52.6%)	133 (50.4%)	—	166 (48.5%)	117 (56.5%)	—	189 (51.5%)	94 (51.6%)	—

*Note:* Bold signifies statistically significant values.

Abbreviations: ECOG PS, Eastern Cooperative Oncology Group performance status; LA-NPC, locally advanced nasopharyngeal carcinoma; N, nodal; P/PDW, platelet-to-platelet distribution width ratio; SII, systemic immune inflammation index; SIRI, systemic inflammation response index; T, tumor.

**Table 3:** Univariate analysis of potential factors associated with PFS and OS.

Variables	PFS			OS		
	Case	MST (m)	*p*	Case	MST (m)	*p*
Age						
<65	509	73.173 ± 1.640	0.537	509	80.139 ± 1.466	0.238
≥65	40	69.529 ± 6.134	—	40	71.729 ± 6.191	—
Gender						
Female	156	70.995 ± 2.892	0.861	156	79.809 ± 2.664	0.893
Male	393	73.121 ± 1.882	—	393	79.669 ± 1.689	—
T stage						
1-2	239	80.291 ± 2.114	**<0.001**	239	84.908 ± 1.924	**0.001**
3-4	310	67.018 ± 2.232	—	310	75.528 ± 2.021	—
N stage						
0-1	66	79.666 ± 3.792	0.050	66	85.396 ± 3.377	0.152
2-3	483	71.741 ± 1.725	—	483	78.812 ± 1.566	—
Clinical stage						
III	400	75.396 ± 1.812	**0.009**	400	81.060 ± 1.636	0.098
IV	149	65.396 ± 3.149	—	149	75.021 ± 2.821	—
ECOG PS						
0	266	72.180 ± 2.281	0.631	266	79.284 ± 1.999	0.789
1–2	283	71.082 ± 2.107	—	283	77.136 ± 2.613	—
P/PDW						
Low	285	79.686 ± 1.970	**<0.001**	285	86.742 ± 1.612	**<0.001**
High	264	65.462 ± 2.423	—	264	72.227 ± 2.233	—
SIRI						
Low	342	78.059 ± 1.857	**<0.001**	342	84.694 ± 1.578	**<0.001**
High	207	58.104 ± 2.375	—	207	69.675 ± 2.774	—
SII						
Low	367	77.532 ± 1.818	**<0.001**	367	83.160 ± 1.588	**0.001**
High	182	59.661 ± 2.720	—	182	70.971 ± 2.933	—

*Note:* Bold signifies statistically significant values.

Abbreviations: ECOG PS, Eastern Cooperative Oncology Group performance status; N, nodal; OS, overall survival; P/PDW, platelet-to-platelet distribution width ratio; PFS, progression-free survival; SII, systemic immune inflammation index; SIRI, systemic inflammation response index; T, tumor.

**Table 4:** Multivariable Cox regression analyses for PFS and OS.

Variables	PFS		OS	
	HR (95% CI)	*p*	HR (95% CI)	*p*
T stage				
1-2	0.539 (0.378–0.768)	**0.001**	0.545 (0.364–0.815)	**0.003**
3-4	—		—	
Clinical stage				
III	0.847 (0.603–1.190)	0.338	—	—
IV	—		—	
P/PDW				
Low	0.544 (0.390–0.759)	**＜0.001**	0.406 (0.268–0.615)	**＜0.001**
High	—		—	
SIRI				
Low	0.688 (0.466–1.017)	0.061	0.525 (0.333–0.827)	**0.006**
High	—		—	
SII				
Low	0.830 (0.555–1.241)	0.365	1.021 (0.641–1.626)	0.930
High	—		—	

*Note:* Bold signifies statistically significant values.

Abbreviations: CI, confidence interval; HR, hazard ratio; OS, overall survival; P/PDW, platelet-to-platelet distribution width ratio; PFS, progression-free survival; SII, systemic immune inflammation index; SIRI, systemic inflammation response index; T, tumor.

## Data Availability

The data analyzed in this study are available from the corresponding author (Yangchun Xie, E-mail: xieyangchun88@csu.edu.cn) on reasonable requests.
